# Modified 5-aminolevulinic acid photodynamic therapy suppresses cutaneous squamous cell carcinoma through blocking Akt/mTOR-mediated autophagic flux

**DOI:** 10.3389/fphar.2023.1114678

**Published:** 2023-03-17

**Authors:** Qingyu Zeng, Jia Liu, Yu Yan, Guolong Zhang, Periru Wang, Haiyan Zhang, Xiaojing Liu, Linglin Zhang, Xiuli Wang

**Affiliations:** School of Medicine, Shanghai Skin Disease Hospital, Institute of Photomedicine, Tongji University, Shanghai, China

**Keywords:** M-PDT, cSCC, autophagic flux, ROS, AKT/mTOR signaling

## Abstract

**Background:** We previously found that modified 5-aminolevulinic acid photodynamic therapy (M-PDT) is painless and effective in cutaneous squamous cell carcinoma (cSCC) treatment, however, the regulatory mechanism of M-PDT in cSCC is still unclear.

**Objective:** To clarify the effect and relevant regulatory mechanism of M-PDT in cSCC.

**Methods:** The cSCC apoptosis was examined by flow cytometry, TUNEL staining and Cleaved-caspase-3 immunofluorescence, respectively. The autophagy-related characterization was detected by monodansylcadaverine (MDC) staining, transmission electron microscopy (TEM), GFP-LC3B autophagic vacuoles localization and mRFP-EGFP tandem fluorescence-tagged LC3B construct, respectively. The expression of autophagy-related proteins and Akt/mTOR signaling molecules were examined by Western blot. ROS generation was measured by DCFH-DA probe.

**Results:** We found that M-PDT induced cSCC apoptosis in a dose-dependent manner, and this result was related to autophagic flux blockage. The phenomenon is confirmed by the results that M-PDT could induce autophagosomes accumulation and upregulate LC3-II and p62 expression. M-PDT elevated co-localization of RFP and GFP tandem-tagged LC3B puncta in cSCC cell, reflecting autophagic flux blockage, and this was confirmed by transmission electron microscopy. Furthermore, we noticed that M-PDT induced accumulated autophagosomes-dependent apoptosis *via* targeting ROS-mediated Akt/mTOR signaling. Suppression of Akt potentiated M-PDT-induced upregulation of LC3-II and p62 levels, whereas Akt activation and ROS inhibition rendered resistance to these events. In addition, we observed that lysosomal dysfunction was involved in M-PDT-triggered accumulated autophagosomes-dependent cSCC apoptosis.

**Conclusion:** Our data demonstrates that M-PDT inhibits cSCC through blocking Akt/mTOR-mediated autophagic flux.

## 1 Introduction

cSCC is a common cutaneous malignant tumor worldwide (Hedberg et al., 2022). Even though cSCC is reported only 5% metastasis and has long-term survival after treatment, the aging and growing population has led to an increasing incidence and mortality rate (Shalhout et al., 2022).

5-aminolevulinic acid photodynamic therapy (ALA-PDT) is based on photosensitizer, light source and oxygen to generate cytotoxic effects on targeted position (Zeng et al., 2022a). ALA-PDT is extensively applied in actinic keratosis (AK), Bowen disease (BD), superficial skin SCC, and other cancerous and precancerous skin diseases (Skaria, 2014). Yet we observed that there is apparent pain in conventional ALA-PDT treatment in clinical, which make patients suspend treatment (Arits et al., 2010; Wang et al., 2017). The pain is a key factor limiting efficacy during ALA-PDT. We previously modified conventional ALA-PDT by reducing the ALA incubation time, and increasing the light dose, then we found that this modified ALA-PDT (M-PDT) not only reduced pain but also had acceptable therapeutic effect in severe acne vulgaris (Zhang et al., 2021), condylomata acuminata (Zhang et al., 2020) and cSCC(Liu et al., 2021; Zeng et al., 2022c). However, the molecular mechanism of M-PDT in cSCC remains unclear.

Autophagy is a conserved degradation system in cells which contains a series of procedures such as double membrane formation and elongation, autophagosomes maturation and fusion with the lysosome (Feng et al., 2015; Zhang et al., 2019a). LC3 is a classic marker of autophagy. LC3 contains two molecular forms, the unconjugated form LC3-I and the conjugated form LC3-II which binds to autophagosomes and associates with the number of autophagosomes (Kabeya et al., 2000; Feng et al., 2015). Therefore, the LC3-II protein level is widely used as a biochemical indicator for assessing the state of autophagy. In addition, autophagy adaptor p62 protein could bind to LC3 and ubiquitinated substrates, and p62 is degraded along with cargo in autophagosomes. The degraded p62 protein level reflects the enhancement of autophagy flux, therefore when autophagy flux is blocked, the p62 protein level accumulates (Zhang et al., 2019a). Thus, p62 protein level is important to evaluate the status of autophagic flux (Sharifi et al., 2015). Several studies have reported that the induction of autophagy commonly occurs after PDT (Martins et al., 2020). However, whether the induced autophagy is beneficial during the PDT treatment seems to be controversial including in ALA-PDT. For example, Liu et al. reported that ALA-PDT induces superoxide anion-dependent autophagic cell death in keloids (Liu et al., 2019), while Zeng et al. (2021) found that autophagy plays a role in promoting survival and inhibiting apoptosis in ALA-PDT treated secondary hyperparathyroidism primary cells. And so far, there is no research on the relationship between M-PDT and autophagy.

In this work, the data showed that M-PDT blocks autophagic flux, leading to autophagosomes-dependent cSCC apoptosis. M-PDT triggers autophagosomes-dependent cSCC cell death by ROS-mediated inhibition of Akt/mTOR signaling pathway. Pharmacological regulation of ROS and Akt could affect M-PDT-induced LC3-II and p62 expression. In addition, we detected that lysosomal dysfunction is involved in M-PDT-induced accumulated autophagosomes-dependent cSCC apoptosis. Our work broaden the molecular mechanism involved in the treatment of M-PDT for cSCC.

## 2 Materials and methods

### 2.1 Materials

N-acetyl-L-cysteine (NAC), Chloroquine diphosphate (CQ), MDC, MK2206, SC79, DCFH-DA probe were obtained from MedChemExpress. 5-Aminolevulinic acid (ALA) was purchased from Sigma.

### 2.2 Cell lines

A431 (ATCC) and SCL-1 (Daixuan Biotech) cSCC cell lines were cultured in DMEM (HyClone) supplemented with 10% FBS (Yeasen) and 100 U/mL penicillin/streptomycin (Yeasen).

### 2.3 Cell viability assay

According to the protocol of manufacturer, 1,000 cells per well were seeded in 96-well plates. After 24 h culture, each well was added 10 μL CCK8 solution (Yeasen) and incubated for 1 h, then cell viability was detected by the microplate reader at 450 nm absorbance.

### 2.4 M-PDT treatment

M-PDT treatment was applied following our published methods (Liu et al., 2021). A431 cells were incubated with ALA (1 mM) in serum-free medium away from light for 1 h at 37°C, then exposed to a red LED light (633 nm) at 15 mW/cm^2^ for 30, 45 and 60 min, the total light dose was 27, 40.5 and 54 J/cm^2^ respectively. As for SCL-1 cells, the illumination time was 20, 22 and 24 min, so the PDT light dose was 18, 19.8 and 21.6 J/cm^2^ respectively.

### 2.5 ROS detection

Before M-PDT irradiation, DCFH-DA probe was added into cell medium for 20 min, and ROS production was observed by an inverted fluorescence microscope.

### 2.6 MDC-labeled autophagic vacuoles

Cells of 5 × 10^5^ per well were seeded in 6-well plate. After different treatments, the cells were exposed to MDC (0.05 mM, 10 min), and washed by PBS, then MDC-marked autophagic vacuoles were detected by an inverted fluorescence microscope.

### 2.7 GFP-LC3B and mRFP-EGFP-LC3B assay

GFP-LC3B and mRFP-EGFP-LC3B lentivirus were purchased from OBiO Technology. After different treatments, cells were fixed with 2% formaldehyde (10 min, room temperature). Autophagosome structure formation was observed by LC3B puncta calculation. Autophagosome maturation was estimated by yellow (accumulation of autophagosomes) and red (autolysosomes) fluorescence structures in cells.

### 2.8 Western blot assay

Western blot analysis was applied as described previously (Zeng et al., 2022b). The following antibodies were used: p-mTOR (Ser2448), mTOR, p-Akt (Ser473), Akt, p-S6K1 (Thr389), S6K1, p-4E-BP1(Thr70), 4E-BP1, p-ULK1 (Ser757), ULK1, Cleaved-PARP, SQSTM1/p62, CTSD, GAPDH, LC3B (Cell Signaling Technology).

### 2.9 Statistical analysis

Data were expressed as the mean value ± standard error (mean ± SE). Student’s t-test for non-paired replicates was used to identify statistically significant differences between treatment means. Group variability and interaction were compared using one-way or two-way analysis of variance followed by Bonferroni’s post-test to compare replicate means. *p* < 0.05 was considered statistically significant.

## 3 Results

### 3.1 M-PDT induces cSCC cell viability suppression

**FIGURE 1:**
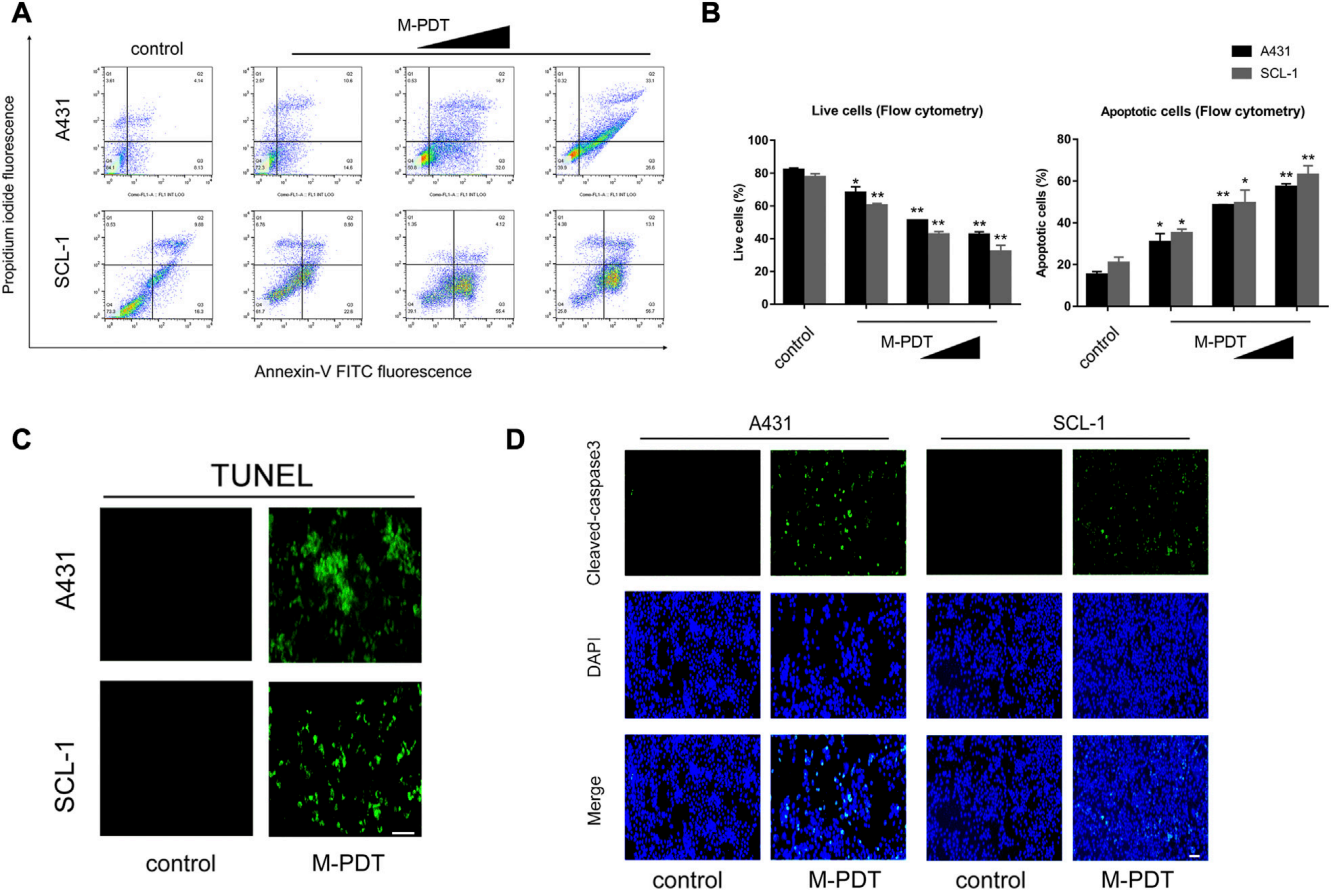
M-PDT induces cSCC cell viability suppression **(A)** The percentages of live and apoptotic cells were measured by FACS using annexin-V-FITC/PI staining. **(B)** Live cells and apoptotic cells in **(A)** were calculated. **(C)** TUNEL staining of cSCC cells. **(D)** After 24 h of M-PDT treatment, immunofluorescence of Cleaved-caspase 3 was detected. The light dose of M-PDT was 54 J/cm^2^ for A431 cells, and 21.6 J/cm^2^ for SCL-1 cells **(C, D)**. *p < 0.05, **p < 0.01, difference vs. control group; the scale bar **(C, D)** is 100 μm. *n* = 3.

**FIGURE 2:**
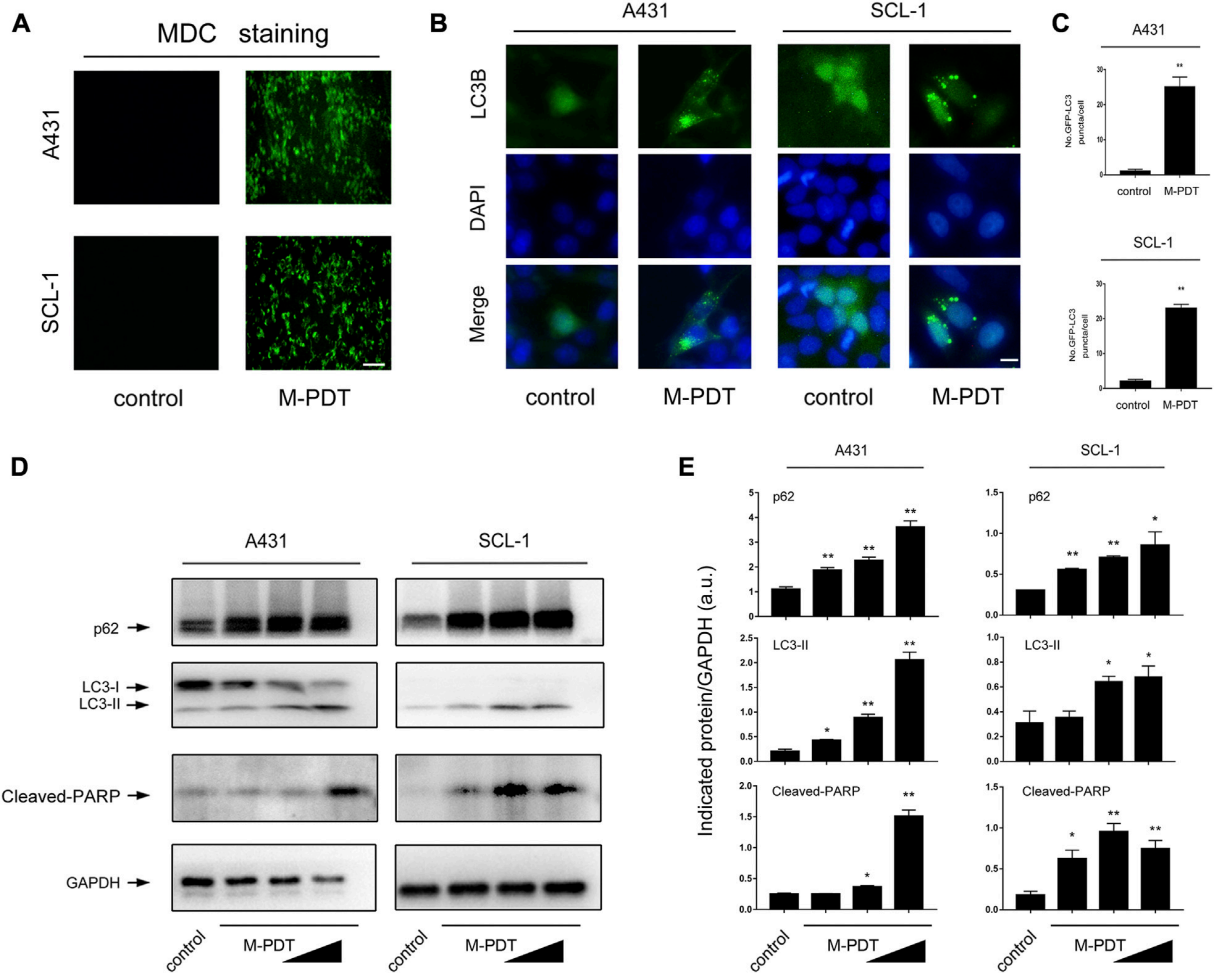
M-PDT induces autophagosomes accumulation and upregulation of LC3-II and p62 expression in cSCC cells **(A)** The cSCC cells were labeled using MDC staining, the scale bar is 100 μm. **(B, C)** Representative GFP-LC3B fluorescence images **(B)** and quantified number for GFP-LC3B puncta **(C)** in cSCC cells, the scale bar is 10 μm. **(D)** Western blot for indicated antibodies. GAPDH is a loading control. Similar results were observed in three independent experiments. **(E)** The relative densities for LC3-II, p62, and Cleaved-PARP were semi-quantified. *p < 0.05, **p < 0.01, difference vs. control group. *n* = 3.

A431 and SCL-1 cells were chosen to study how M-PDT inhibits cSCC cells. We first evaluated apoptosis by Annexin-V-FITC/PI staining. As shown in Figures 1A, B, M-PDT increased the number of apoptotic cells (Q2 and Q3) and decreased the relative number of live cells (Q4) in a concentration-dependent manner. Subsequently, M-PDT significantly elevated the positive cells number of TUNEL (Figure 1C). We next detected the expression of Cleaved caspase-3 and Cleaved PARP in A431 and SCL-1 cells. The immunofluorescence data indicated that M-PDT induced obvious cleavages of caspase-3 in the cSCC cells (Figure 1D). Western blot results in Figures 2D, E revealed that M-PDT increased cleavages of PARP in a concentration-dependent manner in the cSCC cells. Viewed together, the findings clearly demonstrate that M-PDT induces cSCC cell viability suppression.

### 3.2 M-PDT induces autophagosomes accumulation and upregulation of LC3-II and p62 expression in cSCC cells

Several studies have reported that autophagic dysfunction may result in apoptosis (Moscat and Diaz-Meco, 2009; Das et al., 2021). Here we observed that M-PDT significantly increased the accumulation of MDC, a specific autophagic vacuoles marker (Dong et al., 2019) in cSCC cells (Figure 2A). We also confirmed that the LC3-II and p62 protein levels were elevated by M-PDT in a concentration-dependent manner in cSCC cells (Figures 2D, E). To confirm the findings, A431 and SCL-1 cells were infected with Ad-GFP-LC3B, subsequently treated with M-PDT, the number of LC3B puncta was profoundly upregulated compared with control group (Figures 2B, C). The above findings indicate that M-PDT induces autophagosomes accumulation and concomitantly increases protein expression of LC3-II and p62 in cSCC cells, implying that M-PDT could hinder autophagic flux, contributing to subsequent cSCC cell viability suppression.

### 3.3 M-PDT hinders autophagic flux leading to autophagosomes-dependent cSCC cell viability suppression

**FIGURE 3:**
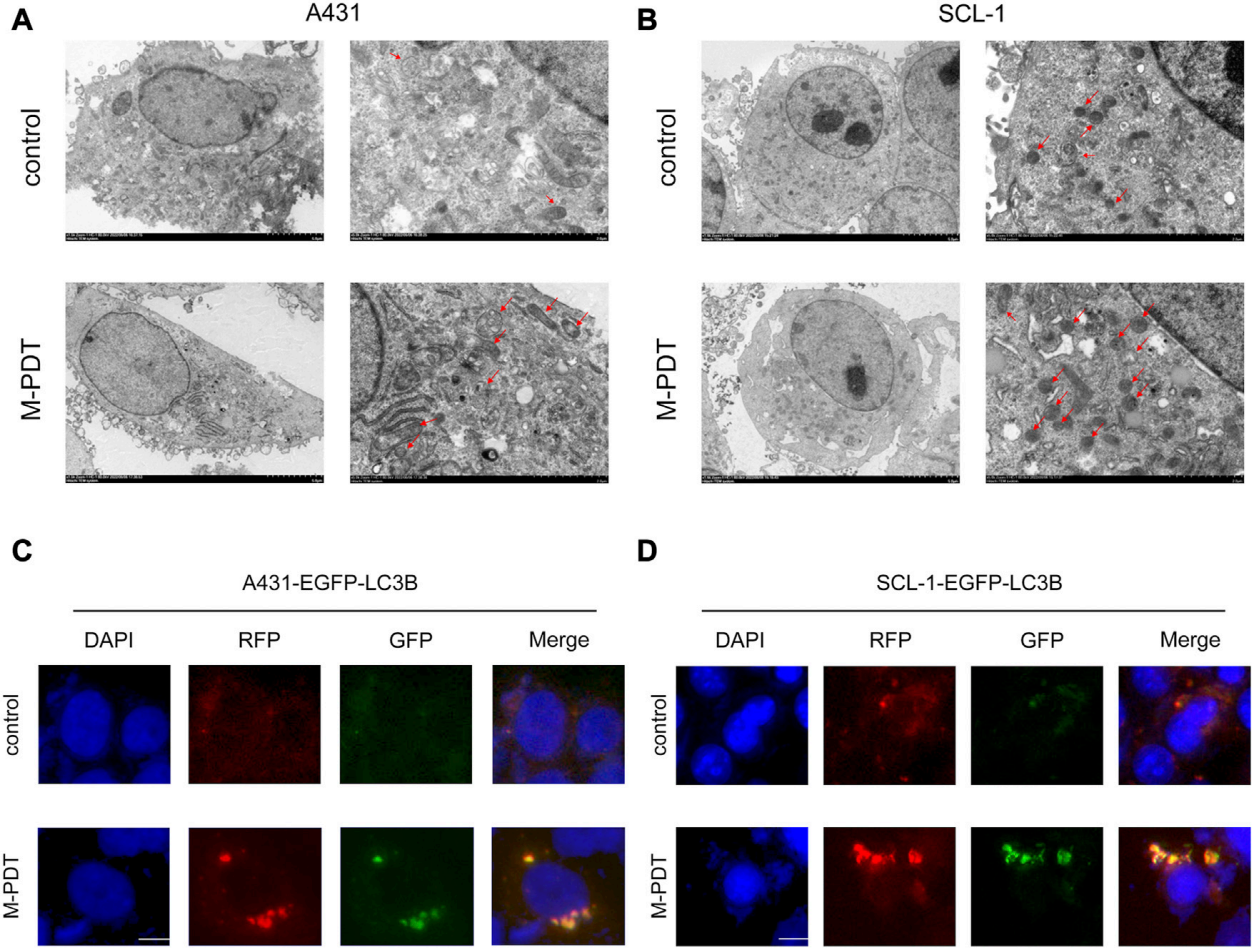
M-PDT impairs autophagic flux of cSCC cells. **(A, B)** Cell structure is detected by transmission electron microscopy, the red arrow shows autophagic vacuoles. **(C, D)** Representative images of fluorescence analysis of autophagic flux in cSCC cells. The scale bar is 10 μm. *n* = 3.

Autophagosome accumulation-induced autophagic flux blockage has been responsible for cell death (Qiu et al., 2014; Yi et al., 2018). Thus, we wanted to know whether M-PDT-induced autophagosome accumulation also impairs autophagic flux and leading to cSCC cell viability suppression. Examination of intracellular structures using TEM demonstrated that M-PDT obviously increased the number of autophagic vacuoles in A431 and SCL-1 cells (Figures 3A, B). Moreover, we measured autophagic flux in A431 and SCL-1 cells expressing mRFP-EGFP tandem fluorescence-tagged LC3B construct. As shown in Figures 3C, D, the cells in the control group showed only slight red-fluorescence structures (autolysosomes), while the cells in the M-PDT-treated groups showed obvious yellow-fluorescence structures (autophagosomes), which exhibits autophagic flux was blocked. The above data support the concept that M-PDT blocks autophagic flux in cSCC cells.

**FIGURE 4:**
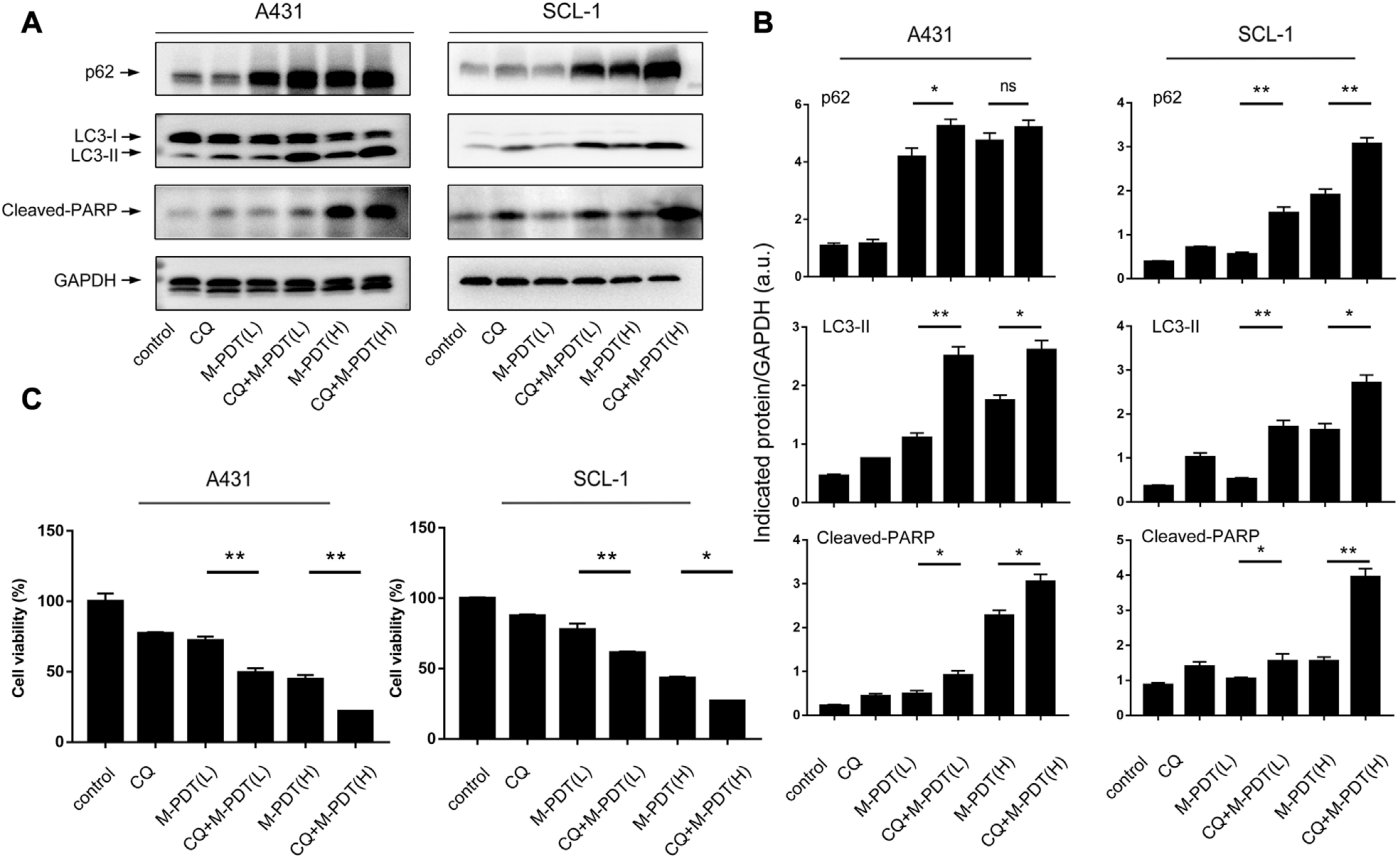
CQ potentiates M-PDT-induced autophagic flux blockage and cSCC cell viability suppression. **(A)** Western blot for indicated antibodies. GAPDH is a loading control. **(B)** The blots for LC3-II, p62, and Cleaved-PARP were semi-quantified. **(C)** Cell viability was detected by CCK8. M-PDT (L) stands for low dose M-PDT, and M-PDT (H) stands for high dose M-PDT. *p < 0.05, **p < 0.01. *n* = 3.

Next, cSCC cells were pretreated with/without CQ (5 μM) for 1 h before M-PDT. CQ could suppress the fusion of autophagosomes and lysosomes. As shown in Figures 4A, B, CQ obviously potentiated the M-PDT-upregulated LC3-II, p62, and Cleaved-PARP protein levels. Furthermore, CCK8 (Figure 4C) results demonstrated that CQ potentiated the inhibition of M-PDT on cell viability. Collectively, the data suggested that M-PDT hinders autophagic flux contributing to autophagosomes-dependent cSCC cell viability suppression.

### 3.4 M-PDT triggers autophagosomes-dependent cSCC cell viability inhibition through ROS-mediated inhibition of Akt/mTOR pathway

**FIGURE 5:**
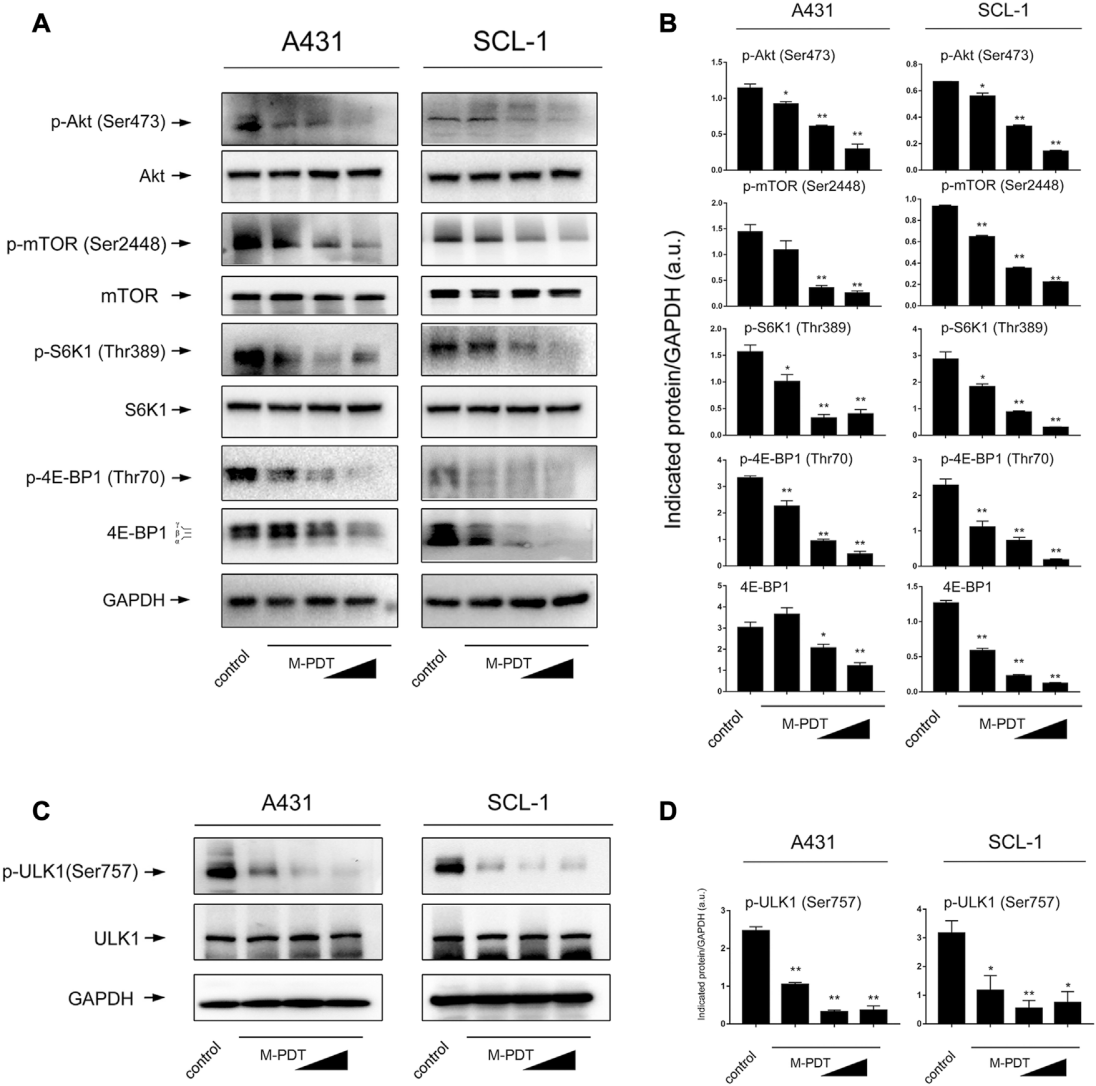
M-PDT inhibits Akt/mTOR signaling pathway in cSCC cells. **(A, C)** Western blot for indicated antibodies. GAPDH is a loading control. **(B, D)** The blots for p-Akt, p-mTOR, p-S6K1, p-4E-BP1,4E-BP1, p-ULK1 were semi-quantified. *p < 0.05, **p < 0.01, difference vs. control group. *n* = 3.

It is known that Akt/mTOR signaling pathway plays an essential role in autophagy regulation (Munson and Ganley, 2015). Therefore, we wondered whether Akt/mTOR pathway is involved in M-PDT-mediated autophagy in cSCC cells. The results in Figures 5A, B showed that M-PDT concentration-dependently suppresses the phosphorylation of Akt, the phosphorylation of mTOR, the phosphorylation of S6K1 and the phosphorylation of 4EBP1 and 4EBP1 expression in cSCC cells, indicating that M-PDT indeed inhibits the Akt/mTOR pathway. The ULK1 is an important regulator in autophagy initiation and mTORC1 induces ULK1 inactivation by phosphorylating ULK1 at Ser757 site, thereby suppressing autophagy (Kim et al., 2011). As observed in Figures 5C, D, we found M-PDT concentration-dependently inhibited ULK1 phosphorylation levels at Ser757 (inactive ULK1 form), which is coincide with Akt/mTOR inhibition (Figures 5A, B).

**FIGURE 6:**
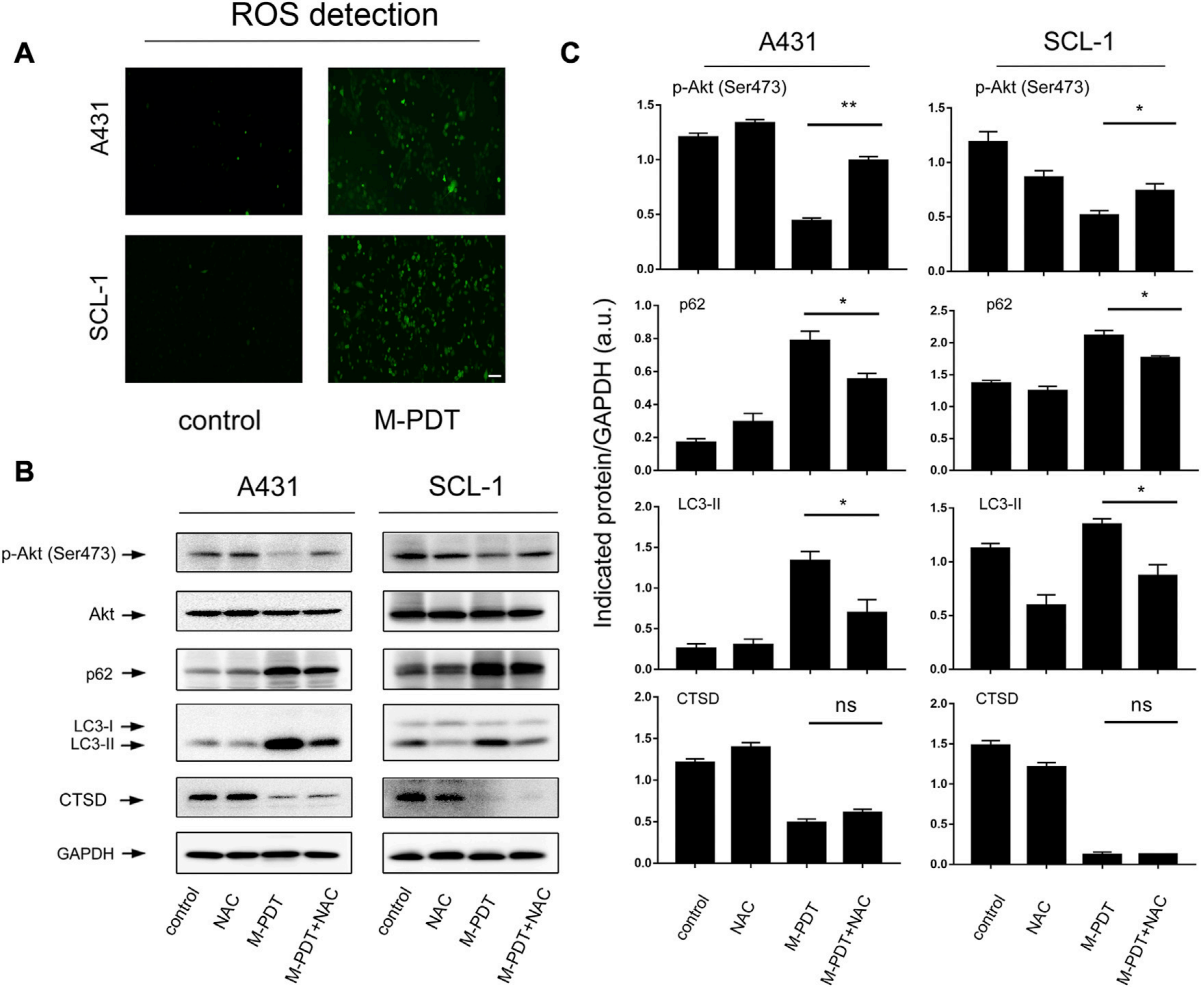
M-PDT impairs autophagic flux through ROS-mediated inhibition of Akt/mTOR pathway in cSCC cells. **(A)** ROS production was measured by DCFH-DA probe, the scale bar is 100 μm. **(B)** Total cell lysates were subjected to Western blotting using indicated antibodies. GAPDH is a loading control. **(C)** The blots for p-Akt, LC3-II, p62, and CTSD were semi-quantified. *p < 0.05, **p < 0.01. *n* = 3.

ROS production is essential for the function of M-PDT (Zeng et al., 2022c). We wanted to know whether M-PDT-induced autophagy is ROS-dependent. ROS generation was detected by DCFH-DA probe in Figure 6A. M-PDT could promote ROS generation of cSCC cells. And NAC (10 μM, pretreatment for 1 h) could significantly inhibit M-PDT-elevated LC3-II and p62 and activate M-PDT-inhibited phosphorylation of Akt in cSCC cells (Figures 6B, C). These results reflect that M-PDT triggers autophagosomes-dependent cSCC cell viability inhibition *via* ROS-dependent inhibition of Akt/mTOR pathway in cSCC cells. CTSD (Cathepsin D) is a specific indicator of lysosomes dysfunction and CTSD is considered as an essential factor for lysosomal proteolytic activity (Di et al., 2021; Hossain et al., 2021). We found that M-PDT obviously decreased the expression of CTSD, while NAC could not rescue M-PDT-suppressed expression of CTSD in A431 and SCL-1 cells (Figures 6B, C). The results imply that M-PDT could induce lysosomal dysfunction in a ROS-independent manner.

### 3.5 Pharmacological regulation of Akt affects M-PDT-induced accumulated autophagosomes-dependent cSCC cell viability suppression

**FIGURE 7:**
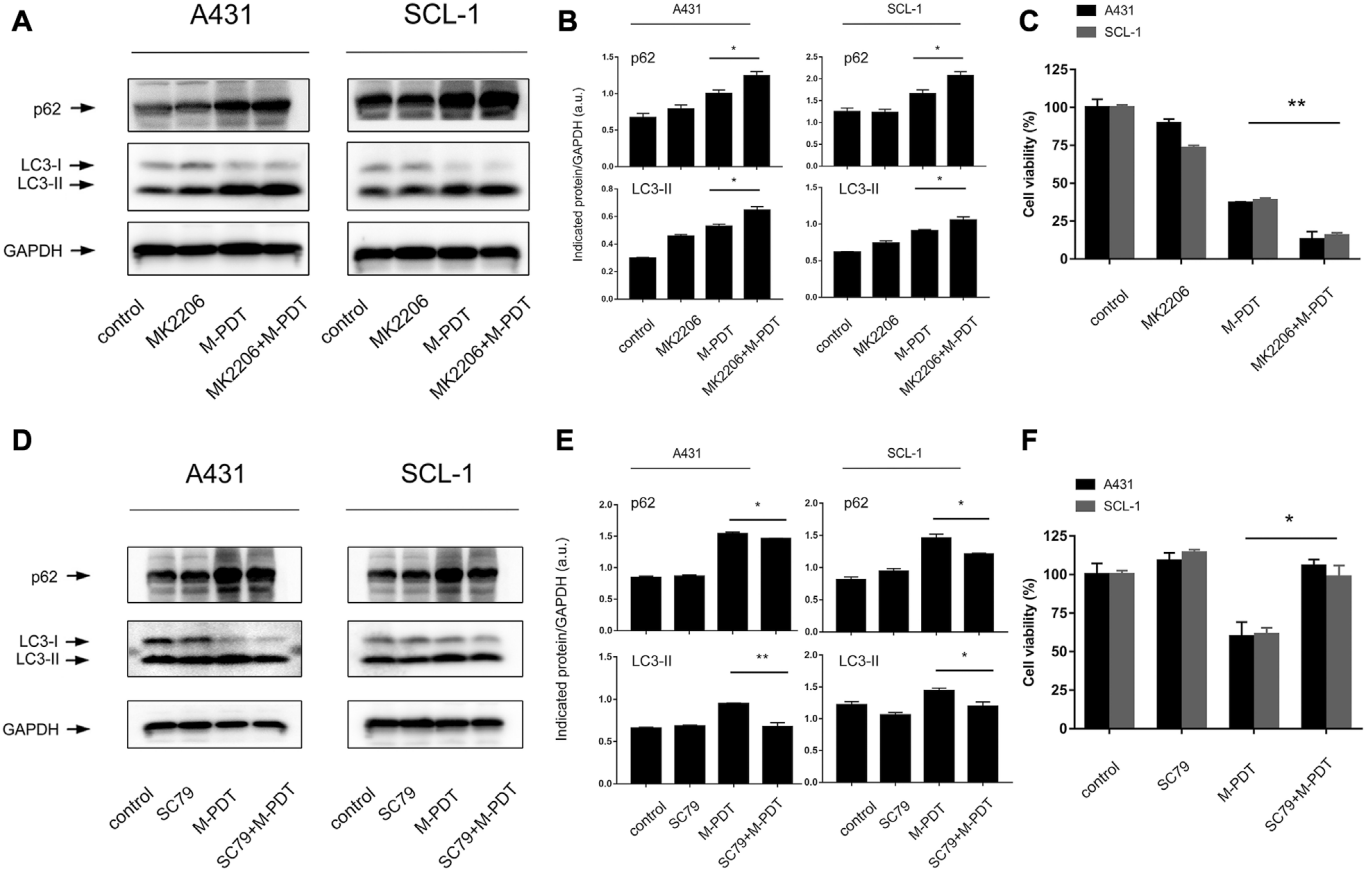
Pharmacological regulation of Akt could affect M-PDT-induced accumulated autophagosomes-dependent cSCC cell viability suppression. **(A, D)** Western blot for indicated antibodies. GAPDH is a loading control. **(B, E)** The relative densities of LC3-II and p62 were semi-quantified. **(C, F)** Cell viability was detected by CCK8. *p < 0.05, **p < 0.01. *n* = 3.

Studies have shown that Akt is an important regulator of autophagic flux as well as cell death (Rodriguez-Hernandez et al., 2018; Dong et al., 2019). A431 and SCL-1 cells were pretreated with or without Akt inhibitor MK2206 (500 nM) for 1 h, and then treated with M-PDT. We found that pretreatment with MK2206 obviously enhanced M-PDT-induced LC3-II, p62 protein levels (Figures 7A, B). In addition, MK2206 also potentiated M-PDT’s inhibition of cell viability in cSCC cells (Figure 7C). To verify the above findings, an Akt agonist, SC79 was employed. As expected, SC79 (5 μM, pretreatment for 1 h) dramatically attenuated M-PDT-induced increase of LC3-II, p62 levels (Figures 7D, E). Consistently, we also observed that SC79 partially prevented M-PDT’s inhibition of cell viability (Figure 7F). Collectively, these data demonstrate that pharmacological regulation of Akt affects M-PDT-induced accumulated autophagosomes-dependent cSCC cell viability suppression.

## 4 Discussion

We previously clarified that M-PDT is painless and effective in cSCC treatment (Liu et al., 2021; Zeng et al., 2022c), but the underlying inhibitory mechanism remains unclear. The relationship between cell death and autophagy has been widely reported (Gao et al., 2020). Even though the subcellular localization and thus the initial targets for photodamage during PDT protocols vary, the induction of autophagy always occurs, independent of the photosensitizer (PS) used (Reiners et al., 2010). The effect of autophagy on the curative outcome of PDT seems to depend on the type of PS, the cell type, the applied light dose, and its concentration (Garg et al., 2015). For example, Shao et al. (2022) reported that the role of Curcumin-PDT-induced autophagy in the regulation of EMT was found to be a promoting effect in lung cancer. Luo et al. (2021) reported that suppression of autophagy elevates apoptosis induced by Ce6-PDT in colon cancer. However, Hyo et al. reported that Pheophorbide a-mediated PDT induces autophagic apoptosis in human skin cancer cells (Yoon et al., 2014). Here, whether the induced autophagy is beneficial during the PDT treatment seems to be controversial.

Our study focused on autophagy involved in the treatment of M-PDT for cSCC. Zeng et al. (2021) reported that CQ enhanced apoptosis induced by ALA-PDT in secondary hyperparathyroidism primary cells. Li et al. found that blockage of autophagy could upregulate the apoptosis rate of HR-HPV-infected HeLa cells after ALA-PDT (Deng et al., 2021). However, Ji et al. (2010) found that ALA-PDT could induce autophagic cell death *via* AMPK and the autophagy inhibitor 3-MA elevated the survival rate of PC12 cell in a dose-dependent manner after ALA-PDT. There is few evidence about the association between ALA-PDT with autophagy in cSCC. Zhang et al. (2019b) reported that 3-MA combined with ALA-PDT could inhibit the proliferation of A431 and A375 cells. Here, we reported that M-PDT suppresses cSCC through blocking Akt/mTOR-mediated autophagic flux. CQ potentiated the inhibition of M-PDT in cSCC. Based on current literatures, we speculate that the differences may be related to the different applied light dose and ALA incubation time, these conditions are exactly the distinctions between traditional ALA-PDT and M-PDT.

Autophagy is a double-edged sword for the cell physiological function (Zhang et al., 2016). If autophagosomes can not to fuse with lysosomes, accumulated autophagosome will block autophagic flux and induce cell death (Button and Luo, 2017; Dong et al., 2019). Due to its characteristic of binding to LC3, the expression level of p62 is an important indicator of autophagic flux (Mizushima et al., 2010). In this study, we showed that M-PDT-induced cSCC cell viability suppression was associated with increase of autophagosomes. Western blot analysis revealed that both LC3-II and p62 were elevated in M-PDT group (Figure 2D). These data suggested that M-PDT triggered autophagic flux blockage. Further evidence was obtained when autophagic flux was detected using mRFP-EGFP tandem fluorescence-tagged LC3 construct in Figure 3. Thus, our results suggest that M-PDT could hinder the fusion of autophagosomes with lysosomes and block autophagic flux in cSCC cells.

Akt/mTOR signaling is a key pathway in regulating autophagy as well as cell viability (Bruntz et al., 2014; Rodriguez-Hernandez et al., 2018). We observed that M-PDT inhibits ROS-mediated Akt/mTOR signaling and hinders autophagic flux, which promotes cSCC cell viability inhibition. Inhibition of Akt with MK2206 strengthened M-PDT-induced upregulation of LC3-II and p62 levels, whereas activate Akt with SC79 rendered resistance to the events. Further experiment elucidated that M-PDT inactivates Akt *via* ROS production. The lysosome is unique organelle to degrade cargos in autophagy, thence lysosomes dysfunction exhibits serious autophagic flux impairment (Qiu et al., 2014). Lysosomes require a full range of proteases to carry out their function. CTSD is an important aspartic protease in the lysosomes (Hossain et al., 2021). Previous study has indicated that CTSD activity change is a specific indicator of lysosomes dysfunction (Ashtari et al., 2016; Schultz et al., 2016). We also found that M-PDT could affect the protein levels of CTSD, this means that the function of the lysosome is also impaired.

In summary, we demonstrated that M-PDT inactivates ROS-mediated Akt/mTOR pathway, which blocks autophagic flux, leading to accumulated autophagosomes-dependent cSCC cell viability suppression. Our results unveil that M-PDT provides a potential solution for the treatment of cSCC and inhibition of autophagy could improve the curative effect of M-PDT.

## 5 Conclusion

**FIGURE 8:**
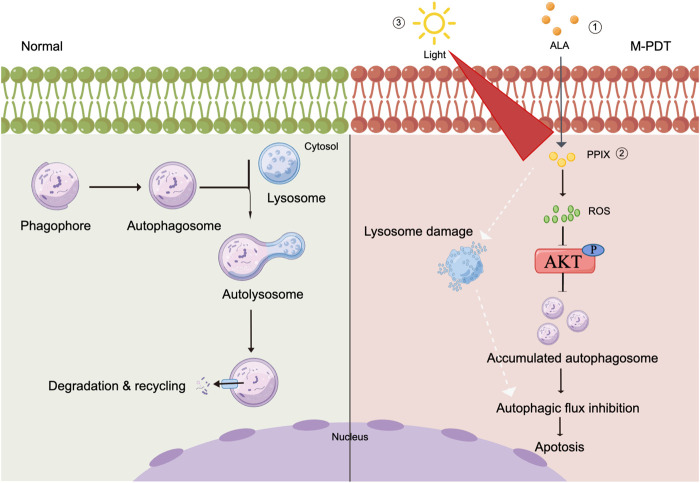
Graphical model demonstrates that M-PDT hinders autophagic flux contributing to cSCC cell viability suppression. M-PDT inhibits ROS-mediated Akt, thereby hinders autophagic flux. Consequently, the accumulated autophagosomes induce cSCC cell viability inhibition. In addition, M-PDT could induce lysosomal dysfunction in a ROS-independent manner. The figure is created by Figdraw.

We have shown that M-PDT inactivates ROS-mediated Akt/mTOR signaling, which blocks autophagic flux, leading to autophagosomes accumulation. Furthermore, the accumulated autophagosomes induce cSCC cell viability inhibition (Figure 8). In addition, M-PDT could induce lysosomal dysfunction in a ROS-independent manner. Our results underline that suppression of autophagic flux is a promising combination with M-PDT against cSCC.

## Data Availability

The raw data supporting the conclusions of this article will be made available by the authors, without undue reservation.

## References

[B1] Arits A. H. van de Weert M. M. Nelemans P. J. Kelleners-Smeets N. W. (2010). Pain during topical photodynamic therapy: Uncomfortable and unpredictable. J. Eur. Acad. Dermatol Venereol. 24, 1452–1457. 10.1111/j.1468-3083.2010.03670.x 20456543

[B2] Ashtari N. Jiao X. Rahimi-Balaei M. Amiri S. Mehr S. E. Yeganeh B. (2016). Lysosomal acid phosphatase biosynthesis and dysfunction: A mini review focused on lysosomal enzyme dysfunction in brain. Curr. Mol. Med. 16, 439–446. 10.2174/1566524016666160429115834 27132795

[B3] Bruntz R. C. Taylor H. E. Lindsley C. W. Brown H. A. (2014). Phospholipase D2 mediates survival signaling through direct regulation of Akt in glioblastoma cells. J. Biol. Chem. 289, 600–616. 10.1074/jbc.M113.532978 24257753 PMC3887188

[B4] Button R. W. Luo S. (2017). The formation of autophagosomes during lysosomal defect: A new source of cytotoxicity. Autophagy 13, 1797–1798. 10.1080/15548627.2017.1358850 28820297 PMC5640195

[B5] Das S. Shukla N. Singh S. S. Kushwaha S. Shrivastava R. (2021). Mechanism of interaction between autophagy and apoptosis in cancer. Apoptosis 26, 512–533. 10.1007/s10495-021-01687-9 34510317

[B6] Deng Z. Chen M. Liu Y. Xu S. Ouyang Y. Shi W. (2021). A positive feedback loop between mTORC1 and cathelicidin promotes skin inflammation in rosacea. EMBO Mol. Med. 13, e13560. 10.15252/emmm.202013560 33734592 PMC8103105

[B7] Di Y. Q. Han X. L. Kang X. L. Wang D. Chen C. H. Wang J. X. (2021). Autophagy triggers CTSD (cathepsin D) maturation and localization inside cells to promote apoptosis. Autophagy 17, 1170–1192. 10.1080/15548627.2020.1752497 32324083 PMC8143247

[B8] Dong X. Zhao R. Li Y. Yu Q. Chen X. Hu X. (2019). Maduramicin inactivation of Akt impairs autophagic flux leading to accumulated autophagosomes-dependent apoptosis in skeletal myoblast cells. Int. J. Biochem. Cell Biol. 114, 105573. 10.1016/j.biocel.2019.105573 31325628 PMC9175263

[B9] Feng Y. Yao Z. Klionsky D. J. (2015). How to control self-digestion: Transcriptional, post-transcriptional, and post-translational regulation of autophagy. Trends Cell Biol. 25, 354–363. 10.1016/j.tcb.2015.02.002 25759175 PMC4441840

[B10] Gao L. Loveless J. Shay C. Teng Y. (2020). Targeting ROS-mediated crosstalk between autophagy and apoptosis in cancer. Adv. Exp. Med. Biol. 1260, 1–12. 10.1007/978-3-030-42667-5_1 32304028

[B11] Garg A. D. Maes H. Romano E. Agostinis P. (2015). Autophagy, a major adaptation pathway shaping cancer cell death and anticancer immunity responses following photodynamic therapy. Photochem Photobiol. Sci. 14, 1410–1424. 10.1039/c4pp00466c 25666525

[B12] Hedberg M. L. Berry C. T. Moshiri A. S. Xiang Y. Yeh C. J. Attilasoy C. (2022). Molecular mechanisms of cutaneous squamous cell carcinoma. Int. J. Mol. Sci. 23, 3478. 10.3390/ijms23073478 35408839 PMC8998533

[B13] Hossain M. I. Marcus J. M. Lee J. H. Garcia P. L. Singh V. Shacka J. J. (2021). Restoration of CTSD (cathepsin D) and lysosomal function in stroke is neuroprotective. Autophagy 17, 1330–1348. 10.1080/15548627.2020.1761219 32450052 PMC8205033

[B14] Ji H. T. Chien L. T. Lin Y. H. Chien H. F. Chen C. T. (2010). 5-ALA mediated photodynamic therapy induces autophagic cell death via AMP-activated protein kinase. Mol. Cancer 9, 91. 10.1186/1476-4598-9-91 20426806 PMC2873441

[B15] Kabeya Y. Mizushima N. Ueno T. Yamamoto A. Kirisako T. Noda T. (2000). LC3, a mammalian homologue of yeast Apg8p, is localized in autophagosome membranes after processing. EMBO J. 19, 5720–5728. 10.1093/emboj/19.21.5720 11060023 PMC305793

[B16] Kim J. Kundu M. Viollet B. Guan K. L. (2011). AMPK and mTOR regulate autophagy through direct phosphorylation of Ulk1. Nat. Cell Biol. 13, 132–141. 10.1038/ncb2152 21258367 PMC3987946

[B17] Liu J. Yan G. Chen Q. Zeng Q. Wang X. (2021). Modified 5-aminolevulinic acid photodynamic therapy (M-PDT) inhibits cutaneous squamous cell carcinoma cell proliferation via targeting PP2A/PP5-mediated MAPK signaling pathway. Int. J. Biochem. Cell Biol. 137, 106036. 10.1016/j.biocel.2021.106036 34217813

[B18] Liu T. Ma X. Ouyang T. Chen H. Xiao Y. Huang Y. (2019). Efficacy of 5-aminolevulinic acid-based photodynamic therapy against keloid compromised by downregulation of SIRT1-SIRT3-SOD2-mROS dependent autophagy pathway. Redox Biol. 20, 195–203. 10.1016/j.redox.2018.10.011 30368039 PMC6205077

[B19] Luo M. Li H. Han D. Yang K. Kang L. (2021). Inhibition of autophagy enhances apoptosis induced by Ce6-photodynamic therapy in human colon cancer cells. Photodiagnosis Photodyn. Ther. 36, 102605. 10.1016/j.pdpdt.2021.102605 34715368

[B20] Martins W. K. Belotto R. Silva M. N. Grasso D. Suriani M. D. Lavor T. S. (2020). Autophagy regulation and photodynamic therapy: Insights to improve outcomes of cancer treatment. Front. Oncol. 10, 610472. 10.3389/fonc.2020.610472 33552982 PMC7855851

[B21] Mizushima N. Yoshimori T. Levine B. (2010). Methods in mammalian autophagy research. Cell 140, 313–326. 10.1016/j.cell.2010.01.028 20144757 PMC2852113

[B22] Moscat J. Diaz-Meco M. T. (2009). p62 at the crossroads of autophagy, apoptosis, and cancer. Cell 137, 1001–1004. 10.1016/j.cell.2009.05.023 19524504 PMC3971861

[B23] Munson M. J. Ganley I. G. (2015). MTOR, PIK3C3, and autophagy: Signaling the beginning from the end. Autophagy 11, 2375–2376. 10.1080/15548627.2015.1106668 26565689 PMC4835211

[B24] Qiu W. Su M. Xie F. Ai J. Ren Y. Zhang J. (2014). Tetrandrine blocks autophagic flux and induces apoptosis via energetic impairment in cancer cells. Cell Death Dis. 5, e1123. 10.1038/cddis.2014.84 24625982 PMC3973245

[B25] Reiners J. J. Jr. Agostinis P. Berg K. Oleinick N. L. Kessel D. (2010). Assessing autophagy in the context of photodynamic therapy. Autophagy 6, 7–18. 10.4161/auto.6.1.10220 19855190 PMC2861993

[B26] Rodriguez-Hernandez M. A. Gonzalez R. de la Rosa A. J. Gallego P. Ordonez R. Navarro-Villaran E. (2018). Molecular characterization of autophagic and apoptotic signaling induced by sorafenib in liver cancer cells. J. Cell Physiol. 234, 692–708. 10.1002/jcp.26855 30132846

[B27] Schultz M. L. Krus K. L. Lieberman A. P. (2016). Lysosome and endoplasmic reticulum quality control pathways in Niemann-Pick type C disease. Brain Res. 1649, 181–188. 10.1016/j.brainres.2016.03.035 27026653 PMC5542880

[B28] Shalhout S. Z. Kaufman H. L. Emerick K. S. Miller D. M. (2022). Immunotherapy for nonmelanoma skin cancer: Facts and hopes. Clin. Cancer Res. 28, 2211–2220. 10.1158/1078-0432.CCR-21-2971 35121622

[B29] Shao L. Zhu Y. Liao B. Wang G. Huang L. Yu L. (2022). Effects of Curcumin-mediated photodynamic therapy on autophagy and epithelial-mesenchymal transition of lung cancer cells. Photodiagnosis Photodyn. Ther. 38, 102849. 10.1016/j.pdpdt.2022.102849 35390521

[B30] Sharifi M. N. Mowers E. E. Drake L. E. Macleod K. F. (2015). Measuring autophagy in stressed cells. Methods Mol. Biol. 1292, 129–150. 10.1007/978-1-4939-2522-3_10 25804753 PMC4460991

[B31] Skaria A. M. (2014). European guidelines for topical PDT part 1 JEADV 2013; 27: 536-544. J. Eur. Acad. Dermatol Venereol. 28, 673. 10.1111/jdv.12258 23981061

[B32] Wang B. Shi L. Zhang Y. F. Zhou Q. Zheng J. Szeimies R. M. (2017). Gain with no pain? Pain management in dermatological photodynamic therapy. Br. J. Dermatol 177, 656–665. 10.1111/bjd.15344 28122416

[B33] Yi H. Wang K. Du B. He L. Ho H. Qiu M. (2018). Aleuritolic acid impaired autophagic flux and induced apoptosis in hepatocellular carcinoma HepG2 cells. Molecules 23, 1338. 10.3390/molecules23061338 29865221 PMC6100546

[B34] Yoon H. E. Oh S. H. Kim S. A. Yoon J. H. Ahn S. G. (2014). Pheophorbide a-mediated photodynamic therapy induces autophagy and apoptosis via the activation of MAPKs in human skin cancer cells. Oncol. Rep. 31, 137–144. 10.3892/or.2013.2856 24253565

[B35] Zeng L. Zou Q. Huang P. Xiong L. Cheng Y. Chen Q. (2021). Inhibition of autophagy with Chloroquine enhanced apoptosis induced by 5-aminolevulinic acid-photodynamic therapy in secondary hyperparathyroidism primary cells and organoids. Biomed. Pharmacother. 142, 111994. 10.1016/j.biopha.2021.111994 34411921

[B36] Zeng Q. Yang J. Ji J. Wang P. Zhang L. Yan G. (2022a). PD-L1 blockade potentiates the antitumor effects of ALA-PDT and optimizes the tumor microenvironment in cutaneous squamous cell carcinoma. Oncoimmunology 11, 2061396. 10.1080/2162402X.2022.2061396 35402079 PMC8986186

[B37] Zeng Q. Yang J. Yan G. Zhang L. Wang P. Zhang H. (2022b). Celastrol inhibits LL37-induced rosacea by inhibiting Ca^2+^/CaMKII-mTOR-NF-κB activation. Biomed. Pharmacother. 153, 113292. 10.1016/j.biopha.2022.113292 35717785

[B38] Zeng Q. Zhou C. Zhang Y. Yan G. Wang X. (2022c). Modified 5-aminolevulinic acid photodynamic therapy reduces pain and improves therapeutic effects in cutaneous squamous cell carcinoma mouse model. Lasers Surg. Med. 54, 804–812. 10.1002/lsm.23516 35066886

[B39] Zhang H. Dong X. Zhao R. Zhang R. Xu C. Wang X. (2019a). Cadmium results in accumulation of autophagosomes-dependent apoptosis through activating Akt-impaired autophagic flux in neuronal cells. Cell Signal 55, 26–39. 10.1016/j.cellsig.2018.12.008 30578829 PMC6378698

[B40] Zhang H. Shi L. Zhang Y. Wang P. Zhang G. Cao Y. (2020). Modified photodynamic therapy to minimize pain in the treatment of condylomata acuminata: A prospective, randomized, self-controlled study. Photodiagnosis Photodyn. Ther. 32, 101915. 10.1016/j.pdpdt.2020.101915 32634656

[B41] Zhang L. Ji Z. Zhang J. Yang S. (2019b). Photodynamic therapy enhances skin cancer chemotherapy effects through autophagy regulation. Photodiagnosis Photodyn. Ther. 28, 159–165. 10.1016/j.pdpdt.2019.08.023 31445100

[B42] Zhang Y. Zhang H. Zhang L. Wang P. Shi L. Zhang G. (2021). Modified 5-aminolevulinic acid photodynamic therapy to reduce pain in the treatment of moderate to severe acne vulgaris: A prospective, randomized, split-face study. J. Am. Acad. Dermatol 84, 218–220. 10.1016/j.jaad.2020.04.146 32376429

[B43] Zhang Z. Miah M. Culbreth M. Aschner M. (2016). Autophagy in neurodegenerative diseases and metal neurotoxicity. Neurochem. Res. 41, 409–422. 10.1007/s11064-016-1844-x 26869037

